# The associations between specific-type sedentary behaviors and cognitive flexibility in adolescents

**DOI:** 10.3389/fnhum.2022.910624

**Published:** 2022-08-12

**Authors:** Jie Cui, Lin Li, Chao Dong

**Affiliations:** ^1^College of Physical Education, Shanghai Normal University, Shanghai, China; ^2^College of Physical Education and Health, East China Normal University, Shanghai, China; ^3^Key Laboratory of Adolescent Health Assessment and Exercise Intervention of Ministry of Education, East China Normal University, Shanghai, China; ^4^College of Physical Education and Health, Shanghai University of International Business and Economics, Shanghai, China

**Keywords:** cognitive flexibility, executive function, sedentary behavior, recreational screen-based sedentary behavior, educational sedentary behavior, adolescents

## Abstract

**Background:** The prevalence of sedentary behavior in adolescents has aroused social attention. The association between sedentary behavior and cognitive flexibility remains unclear, and it may vary depending on the type of sedentary behavior. This study aimed to investigate the associations between specific-type sedentary behaviors and cognitive flexibility in adolescents.

**Method:** A total of 700 Chinese adolescents aged 10–15 years were recruited. The self-report questionnaire was used to assess total sedentary time, recreational screen-based sedentary time, and educational sedentary time. The More-odd shifting task was used to assess cognitive flexibility.

**Results:** The correlation analysis showed that recreational screen-based sedentary time was negatively correlated with cognitive flexibility, whereas educational sedentary time was positively correlated with cognitive flexibility. The regression analysis also further revealed that a significantly negative association between recreational screen-based sedentary time and cognitive flexibility, while a significantly positive association existed between educational sedentary time and cognitive flexibility.

**Conclusion:** The findings shown that the association between recreational screen-based sedentary behavior and cognitive flexibility differs from educational sedentary behavior in adolescents, providing new ideas for a more comprehensive understanding of the association between sedentary behavior and cognitive flexibility in adolescents.

## Introduction

Sedentary behavior in children and adolescents has become a global issue, attracting the attention of governments and health organizations worldwide (Graf et al., 2017; Zhang et al., 2017; World Health Organization, 2020). Sedentary behavior, distinct from physical inactivity (Ng and Popkin, 2012), is defined as any waking behavior characterized by an energy expenditure ≤1.5 metabolic equivalents while sitting, reclining, or lying (Tremblay et al., 2017). Sedentary behavior has been related to a variety of adverse health outcomes in recent years (Carson et al., 2016; Van Ekris et al., 2016), including obesity (Chen et al., 2016), increased all-cause mortality (Ekelund et al., 2019), and cardiovascular disease (Vaisto et al., 2019), implying that it is the new and major risk factor for children’s and adolescents’ health (Owen et al., 2020). Similarly, the negative impact of sedentary behavior may be found in the cognitive dimension (Carson et al., 2015; Falck et al., 2017). The executive function, a higher-order cognitive process, becomes the center of attention. Executive function refers to a set of top-down mental processes required for goal-directed behaviors and is linked to physical and psychological well-being as well as academic achievement in children and adolescents (Diamond, 2013). Surprisingly, the associations between sedentary behavior and executive function in children and adolescents are not well understood (Li et al., 2022). The findings of existing studies are mixed. Some studies identified a negative association between sedentary behavior and executive function (Van der Niet et al., 2015; Zeng et al., 2021), while others showed a positive association (Aadland et al., 2017; Wickel, 2017), and yet another reported no association (Mora-Gonzalez et al., 2019).

The type of sedentary behavior may be one of the major explanations for the inconsistent results of the existing studies. Sedentary behavior can be classified into several types based on the activity’s content and purpose, such as recreational screen-based sedentary behavior (i.e., watching TV or playing computer), educational sedentary behavior (i.e., doing homework), and so on (Hardy et al., 2007). In this field, recreational screen-based sedentary behavior has received the most attention. According to the review study, recreational screen-based sedentary behavior may be detrimental to adolescents’ executive function (Li et al., 2022). Compared to children and adolescents in other countries, Chinese children and adolescents devote more time to educational sedentary activities (i.e., reading, writing, or drawing; Duan et al., 2015; Guo et al., 2017). More importantly, a study found that sedentary time spent doing homework at age 7 years was positively associated with cognition at age 11 years, whereas sedentary time spent watching television was negatively associated with cognition, although the association was attenuated to the null after controlling for baseline cognition (Aggio et al., 2016), indicating that the type of sedentary behavior may be an important factor in understanding the association between sedentary behavior and cognition in adolescents. However, existing research tends to focus on total sedentary time rather than specific sedentary behaviors (Wickel, 2017; Mora-Gonzalez et al., 2019; Santiago-Rodriguez et al., 2022). More research is needed to build on the growing body of evidence linking type-specific sedentary behaviors (Owen et al., 2020), particularly recreational screen-based sedentary behavior and educational sedentary behavior.

In addition, it is well-known to all that executive function includes inhibitory control, working memory, and cognitive flexibility (Duncan et al., 1997; Miyake et al., 2000). Total sedentary time measured with a hip-mounted accelerometer was associated with poor inhibition but not working memory and cognitive flexibility (Van der Niet et al., 2015), implying that the effects of sedentary behavior on three core components of executive function may differ. More studies often viewed executive function as a single structure or concentrated on inhibitory control and working memory (López-Vicente et al., 2017; Horowitz-Kraus et al., 2021), while few explored the association between sedentary behavior and cognitive flexibility in adolescents. Cognitive flexibility is defined as the ability to adjust one’s thoughts and behaviors in response to changing circumstances and goals (Garon et al., 2008), and it could be developed into late childhood (Chevalier et al., 2012). Cognitive flexibility, due to its complex structure, which includes inhibitory control and working memory, is likely to act as a requirement for the use of more cognitively demanding strategies in children’s lives and studies (Richter and Yeung, 2012; Stokes et al., 2013; Hanks and Summerfield, 2017). Therefore, it is critical to explore the association between sedentary behavior and cognitive flexibility in adolescents.

Hence, in contrast to the previous work that focused solely on sedentary time, this current study investigates the associations between specific-type sedentary behaviors and cognitive flexibility in adolescents, examining whether the association between sedentary behavior and cognitive flexibility in adolescents varies by type. Given the high prevalence of recreational screen-based and educational sedentary behaviors in Chinese adolescents (Duan et al., 2015; Guo et al., 2017), this study investigates respectively the associations between total sedentary time, recreational screen-based sedentary behavior, educational sedentary behavior, and cognitive flexibility in adolescents. It was hypothesized that the association between recreational screen-based sedentary behavior and cognitive flexibility in adolescents would differ from the association between educational sedentary behavior and cognitive flexibility.

## Materials and Methods

### Participants

In the current study, 700 healthy adolescents aged 10–15 years were initially recruited in China’s urban areas. The predetermined inclusion criteria were as follows: (1) right-handedness; (2) no psychotropic drug use; (3) no mental or physical disability; (4) normal or corrected-to-normal vision; and (5) no emotional or behavioral problems. The Strength and Difficulty Questionnaire (child version; SDQ) with good reliability and validity was used to assess adolescents’ emotional problems, conduct problems, hyper-activity-inattention, peer problems, and prosocial behavior to ensure they had no emotional or behavioral problems (Goodman, 2001). This study excluded adolescents with SDQ scores greater than 15. Finally, data from 630 adolescents (329 boys) were included in the final analysis after excluding participants with missing information about sedentary behavior or cognitive flexibility, as well as those who did not meet the inclusion criteria. The study protocol was approved by the Institutional Review Board of the East China Normal University and all study procedures met the guideline of the Declaration of Helsinki (No. HR047-2018). After completing the entire experiment, each participant signed an informed consent and received a payment of 50 RMB.

### Procedures

First, self-administered questionnaires were used to collect socio-demographic information (age, sex, grade, and ethnicity). The height and body weight of adolescents were measured using a standardized height measure and scale, with the participants standing bare feet and wearing light clothing. The body mass index (BMI) was calculated as weight in kilograms divided by height in meters squared (BMI= weight (kg) ÷ height (m)^2^). To ensure the accuracy and reliability of the study data, researchers will introduce the questionnaire items in detail, as well as the regulations and operation steps of the cognitive flexibility task for adolescents, prior to the formal test.

Second, under the supervision of a teacher and a researcher, all participants carefully fill out the questionnaires in a clear and calm classroom. To ensure that participants complete these questionnaires honestly and in accordance with their actual situation, the researcher declares before the questionnaires begin that they have no bearing on academic performance.

Finally, all participants complete the More-odd shifting task in a clear and quiet computer classroom. Before the test, the researcher instructs participants to respond to the task as quickly as possible by pressing a button.

### Sedentary behavior

Adolescents reported total sedentary time, recreational screen-based sedentary time, and educational sedentary time using a questionnaire adapted from the Adolescent Sedentary Activities Questionnaire (ASAQ) developed by Hardy et al. (2007). In Chinese children and adolescents, the Chinese vision of ASAQ has good internal consistency (overall Cronbach’ α Coefficient = 0.729) and construct validity (Guo, 2016). This questionnaire also has acceptable test-retest reliability (*r* = 0.192–0.815) for participants in this study. Participants were asked to think about a typical week and report how much time they usually spent on all sedentary activities before and after school on each weekday and weekend. The total sedentary time was calculated by averaging the time spent each day on all sedentary activities. The recreational screen-based sedentary time was calculated using the average time spent on four of all sedentary activities for entertainment (watching television/videos/DVDs and movies, and playing computers/Ipad/phone). The educational sedentary time was calculated using the average time spent on three of all sedentary activities for learning (study with/without computer, and tutoring).

### Physical activity

Physical activity was measured using the Physical Activity Questionnaire for older children and adolescents (PAQ), which was developed by Kowalski et al. (2004) and updated by Guo (2016). This questionnaire has been validated in China for use with children and adolescents, and it has adequate reliability (Cronbach’s *α* = 0.82) and validity (Guo, 2016). This questionnaire was divided into three sections. The first section of the questionnaire was intended to determine how frequently adolescents participate in various sports such as skipping rope and basketball; the second section was intended to determine the adolescents’ physical activity during various periods such as after school and physical education; and the third section was intended to determine the average frequency of physical activity over the previous 7 days. Finally, the average of three sections was calculated and used to describe adolescents’ level of physical activity. The higher the score, the greater the level of physical activity there is. The level of physical activity was classified as high (the score > 3), medium (3 ≥ the score > 2), and low (the score ≤ 2; Chen et al., 2008).

### Cognitive flexibility

Cognitive flexibility was measured using the More-odd shifting task (Chen et al., 2014). The More-odd shifting task consists of a series of numeric digits displayed in the center of the screen, ranging from either 1 to 4 or 6 to 9. All digits were displayed for 1,500 ms, and the ISI was set to 1,000 ms. There were three types of blocks, each of which ran twice. For A block in which the digits were printed in white, participants were required to indicate whether the presented digit was larger than or less than five by pressing the “C” or the “M” key respectively. For B block in which the digits were printed in red, participants were asked to indicate whether the presented digit was odd or even by pressing the “C” or the “M” key respectively. For C block included both A- and B-type trials (16 trials each), with the trials switching from one to the other every two trials. Participants were required to press the “C” or “M” key to identify whether the digit was larger or less than five when the digit was presented in white, and whether the digit was odd or even when the digit was presented in red. The experiment lasted 480 s in total. The accuracy of the heterogeneous (C blocks), the accuracy of the homogeneous (A and B blocks) blocks, as well as the difference in reaction time between the heterogeneous and homogeneous blocks, were recorded and utilized to develop cognitive flexibility indexes.

### Statistical analysis

The data were analyzed using statistical software (SPSS Version 26.0, IBM Corporation, Somers, NY, USA), with a significance level accepted as *p* < 0.05. All descriptive data (age, height, weight, BMI, physical activity, sedentary behavior, and cognitive flexibility) were reviewed and presented as mean ± SD to summarize the participants’ characteristics. The Shapiro-Wilk test was used to assess the normal distribution of sedentary behavior (Shapiro and Wilk, 1965). Then, the Mann-Whitney U was used to calculate the gender difference (boys vs. girls) in sedentary behavior, and the effect size was calculated as the Cohen’s d (Lenhard and Lenhard, 2016). Pearson’s correlation analysis was used to assess the correlations between sedentary behavior (total sedentary time, recreational screen-based sedentary time, and educational sedentary time) and cognitive flexibility (the accuracy and difference in reaction time of the More-odd shifting task). Hierarchical linear regressions analysis was performed to evaluate respectively the association between each independent variable (total sedentary time, recreational screen-based sedentary time, and educational sedentary time) and each cognitive outcome (the accuracy of the heterogeneous blocks, the accuracy of the homogeneous blocks, the difference in the average reaction time between the heterogeneous and the homogeneous blocks in the More-odd shifting task), while adjusting for covariates (age, sex, BMI, and physical activity).

## Results

### Descriptive statistics analysis

The descriptive characteristics of sedentary behavior and cognitive flexibility were presented in Table 1 and Table 2. In terms of total sedentary time, the average time per day was 5.753 (SD = 2.367) hours, and there was no statistically significant difference between girls and boys (*p =* 0.479). In terms of recreational screen-based sedentary behavior, the average time per day was 1.438 (SD = 1.500) hours. Furthermore, there was a minor but significant gender difference (*p* < 0.049, Cohen’s *d* = 0.158), with boys spending considerably more time on recreational screen-based sedentary behavior than girls. In terms of educational sedentary behavior, the average time per day was 2.437 (SD = 1.451) hours, with no significant difference between girls and boys (*p =* 0.796). In terms of physical activity, the average score was 2.325 (SD = 0.879), indicating that adolescents’ physical activity is at a medium level.

**Table 1:** The descriptive characteristics of the participants’ demographic and sedentary behaviors (Mean ± SD).

	**All (*N* = 630)**	**Boys (*N* = 301)**	**Girls (*N* = 329)**
Age (year)	12.019 ± 1.572	11.893 ± 1.546	12.134 ± 1.589
Height (m)	1.519 ± 0.129	1.518 ± 0.144	1.519 ± 0.113
Weight (kg)	45.025 ± 12.866	46.541 ± 14.528	43.638 ± 10.970
BMI (kg/m^2^)	19.217 ± 3.420	19.799 ± 3.666	18.684 ± 3.089
Physical activity	2.325 ± 0.879	2.344 ± 0.880	2.308 ± 0.879
TST (h/d)	5.753 ± 2.367	5.827 ± 2.412	5.685 ± 2.327
Weekday TST (h/d)	4.746 ± 2.172	4.812 ± 2.230	4.687 ± 2.119
Weekend TST (h/d)	8.121 ± 3.631	8.222 ± 3.779	8.029 ± 3.494
SST (h/d)	1.438 ± 1.500	1.551 ± 1.610	1.336 ± 1.386
Weekday SST (h/d)	1.012 ± 1.264	1.037 ± 1.386	0.990 ± 1.143
Weekend SST (h/d)	2.494 ± 2.559	2.822 ± 2.706	2.193 ± 2.382
EST (h/d)	2.437 ± 1.451	2.500 ± 1.553	2.380 ± 1.351
Weekday EST (h/d)	2.211 ± 1.398	2.318 ± 1.533	2.113 ± 1.256
Weekend EST (h/d)	3.003 ± 2.347	2.953 ± 2.410	3.049 ± 2.290

BMI, Body Mass Index; TST, total sedentary time; SST, recreational screen-based sedentary time; EST, educational sedentary time; h/d, hour per day.

**Table 2:** The descriptive characteristics of the participants’ cognitive flexibility (Mean ± SD).

	**All (*N* = 630)**	**Boys (*N* = 301)**	**Girls (*N* = 329)**
AB.ACC (%)	0.876 ± 0.085	0.881 ± 0.087	0.871 ± 0.083
C.ACC (%)	0.857 ± 0.075	0.861 ± 0.076	0.854 ± 0.074
Mos.RT (ms)	202.048 ± 101.189	197.116 ± 103.675	206.561 ± 98.802

AB.ACC, the accuracy of the homogeneous blocks in the More-odd shifting task; C.ACC, the accuracy of the heterogeneous blocks in the More-odd shifting task; Mos.RT, the difference in the average reaction time between the heterogeneous and the homogeneous blocks in the More-odd shifting task.

### Correlation analysis

The recreational screen-based sedentary time was negatively correlated with both the accuracy of the heterogeneous (*r* = −0.134, *p* = 0.001) and homogeneous blocks (*r* = −0.181, *p* < 0.0001), but positively correlated with difference in reaction time between the heterogeneous and the homogeneous blocks (*r* = 0.09, *p* = 0.023); On weekdays, recreational screen-based sedentary time was negatively correlated with the accuracy of the heterogeneous (*r* = −0.155, *p* < 0.001) and homogeneous blocks (*r* = −0.168, *p* < 0.0001). On weekends, it was negatively correlated with the accuracy of the heterogeneous (*r* = −0.082, *p* = 0.038) and homogeneous blocks (*r* = −0.166, *p* < 0.001), while it was positively correlated with difference in reaction time between the heterogeneous and the homogeneous blocks (*r* = 0.093, *p* = 0.019).

The educational sedentary time was both positively correlated with the accuracy of the heterogeneous blocks (*r* = 0.125, *p* = 0.002) and the accuracy of the homogeneous blocks (*r* = 0.127, *p* = 0.001). On weekdays, educational sedentary time was positively correlated with the accuracy of the homogeneous blocks (*r* = 0.107, *p* = 0.007). On weekends, educational sedentary time was positively correlated with both the accuracy of the heterogeneous blocks (*r* = 0.16, *p* < 0.001) and the accuracy of the homogeneous blocks (*r* = 0.115, *p* = 0.004).

There was no correlation between total sedentary time and any of the More-odd shifting task measures (*p* > 0.05).

### Regression analysis

The results of the regression analysis assessing the association between recreational screen-based sedentary time and cognitive flexibility were presented in Table 3. After controlling for age, sex, BMI, and physical activity, recreational screen-based sedentary time was significantly inversely related to the accuracy of heterogeneous blocks (*β* = −0.103, *p* = 0.019, 95% confidence interval (CI) [−0.009, −0.001]); Similarly, on weekdays, it also was significantly inversely related to the accuracy of heterogeneous blocks (*β* = −0.127, *p* = 0.003, 95%CI [−0.012, −0.003]).

**Table 3:** Regression analysis for the associations between sedentary behaviors and cognitive flexibility (*N* = 630).

	**R^2^**	**R^2^_adj_**	***F*_change_**	***P***	**B**	**β**	**95% CI**	
							lower limits	upper limits
AB.ACC								
TST	0.220	0.213	0.092	0.762	0	0.011	−0.002	0.003
SST	0.219	0.213	0.004	0.949	0	0.002	−0.004	0.004
EST	0.220	0.213	0.213	0.644	0.001	0.017	−0.003	0.005
C.ACC								
TST	0.018	0.011	0.090	0.764	0	−0.012	−0.003	0.002
SST	0.027	0.019	5.562	0.019*	−0.005	−0.103	−0.009	−0.001
EST	0.028	0.020	6.037	0.014*	0.005	0.100	0.001	0.009
Mos.RT								
TST	0.039	0.032	2.049	0.153	2.409	0.056	−0.896	5.714
SST	0.037	0.029	0.582	0.446	2.226	0.033	−3.506	7.957
EST	0.039	0.031	1.784	0.182	3.762	0.054	−1.770	9.294

TST, total sedentary time; SST, recreational screen-based sedentary time; EST, educational sedentary time; CI, confidence interval; AB.ACC, the accuracy of the homogeneous blocks in the More-odd shifting task; C.ACC, the accuracy of the heterogeneous blocks in the More-odd shifting task; Mos.RT, the difference in the average reaction time between the heterogeneous and the homogeneous blocks in the More-odd shifting task; R^2^, determination coefficient of the regression; R^2^adj, the adjusted R^2^ from the regression analysis model; *p < 0.05.

The results of the regression analysis assessing the association between educational sedentary time and cognitive flexibility were presented in Table 3. After controlling for age, sex, BMI, and physical activity, educational sedentary time was found to be significantly positively associated with the accuracy of heterogeneous blocks in the More-odd shifting tasks (*β* = 0.1, *p* = 0.014, 95% CI [0.001, 0.009]). Similarly, on weekends, it also was found to be significantly positively associated with the accuracy of heterogeneous blocks in the More-odd shifting tasks (*β* = 0.143, *p* < 0.001, 95% CI [0.002, 0.007]).

The results of the regression analysis assessing the association between total sedentary time and cognitive flexibility were presented in Table 3. No associations were observed between total sedentary time and any measures of the More-odd shifting task (*p* > 0.05) after controlling for age, sex, BMI, and physical activity.

## Discussion

In the current study, we examined whether the association between sedentary behavior and cognitive flexibility differed by type in adolescents. We found innovatively that the association between recreational screen-based sedentary time and cognitive flexibility differs from the association between educational sedentary time and cognitive flexibility. To be specific, recreational screen-based sedentary behavior was found to be negatively associated with cognitive flexibility, whereas educational sedentary behavior was found to be positively associated with cognitive flexibility in adolescents. These findings supported our hypothesis.

Previous studies have demonstrated that recreational screen-based sedentary behavior has a negative impact on adolescents’ cognition (Swing et al., 2010; Weis and Cerankosky, 2010). Furthermore, according to a recent systematic review study, screen-based sedentary behavior in children and adolescents may be negatively associated with executive function (Li et al., 2022). Our findings in this study back up previous studies and reviews by demonstrating a significantly negative association between recreational screen-based sedentary time and cognitive flexibility in adolescents, particularly on weekdays. There could be several explanations for these negative associations. First, recreational screen-based sedentary behavior (i.e., watching TV) frequently provides children and adolescents with intense and stimulating sensory experiences, leading to children spending more time on such behaviors and significantly crowding out time spent on cognitive development-promoting behaviors (i.e., do homework; Koolstra et al., 1997). Second, screen exposure may be related to inattentive symptoms (Zivan et al., 2019), slow processing of cognitive resource, and poor memory ability (Horowitz-Kraus et al., 2021), all of which can impair cognitive flexibility. Third, new neuroimaging studies have revealed that the increased recreational screen-based sedentary behavior was associated with decreased gray matter volume in the frontal lobe (Zavala-Crichton et al., 2020) and decreased fractional anisotropy in white matter tracts related to EF (Hutton et al., 2020). As we all know, the frontal lobe is an important brain area responsible for executive functions such as cognitive flexibility (Pribram, 1976).

Educational sedentary behavior is extremely common in Chinese children and adolescents, owing to increased social and cultural pressures associated with academic performance (Tudor-Locke et al., 2003; Cui et al., 2011). However, only a few studies have explored the association between educational sedentary behavior (i.e., doing homework) and cognitive function in children and adolescents (Aggio et al., 2016; Lizandra et al., 2016; Hunter et al., 2018). Building on prior studies, the current study, in greater depth, investigated the association between educational sedentary behavior and cognitive flexibility. Our findings showed that educational sedentary behavior was positively associated with cognitive flexibility in adolescents, particularly on weekends. On the one hand, these findings could be explained by the high-level cognitive engagement associated with this type of behavior. Adolescents’ cognitive development can be boosted by engaging in cognitively active sedentary behavior such as reading and learning (Sweetser et al., 2012). Sedentary behaviors related to academic skills (i.e., reading and writing) have been shown to be positively associated with cognitive function (Haapala et al., 2014); on the other hand, these findings could also be explained by functional changes in the brain area that supports executive function. Evidence from brain imaging study shows that reading was found to be positively correlated with increased functional connectivity between the visual word form area and regions supporting executive function and cognitive control (i.e., inferior prefrontal gyrus; Horowitz-Kraus and Hutton, 2018).

The current study extends our understanding of the association between sedentary behavior and cognitive flexibility. These novel findings in the current study suggest that the association between recreational screen-based sedentary behavior and cognitive flexibility differs from educational sedentary behavior, and they provide preliminary evidence for the association between sedentary behavior and cognitive flexibility in adolescents varies by type. These findings also suggest that, with the exception of sedentary time, the type of sedentary behavior may have an impact on the association between sedentary behavior and cognitive flexibility, which opens up new avenues for future research. Furthermore, this study discovered that the association between sedentary behavior and cognitive flexibility differs on a weekday vs. weekends, implying that we should limit adolescents’ recreational screen-based sedentary time on weekdays to guarantee healthy cognitive development. However, it is worth noting that these correlations in the current study were statistically significant but small, implying that more research is needed in the future to explore and validate these ideas.

The current study has several limitations that should be mentioned. First, the questionnaire used in this study can well investigate recreational screen-based sedentary behavior, educational sedentary behavior, and total sedentary time. This self-report questionnaire, however, may have some subjective variation. In addition, we did not recruit children from primary school (grades 1–3), because children in grades 1–3 of primary school have a low cognitive level and may not be able to accurately understand the items in these self-report questionnaires. Second, given Chinese adolescents’ sedentary status, this study only surveyed recreational screen-based sedentary behavior and educational sedentary behavior. To fully understand the associations between sedentary behavior and cognitive flexibility in adolescents, researchers should investigate other types of sedentary activity (such as travel sedentary behavior and social sedentary behavior). Lastly, because this is a cross-sectional study rather than a longitudinal cohort study, it is impossible to determine the causal associations between sedentary behavior and cognitive flexibility. Clearly, longitudinal investigations will be required in future studies to explore and validate the deeper associations between sedentary activity and cognitive flexibility in adolescents.

## Conclusion

In summary, our findings show that the association between recreational screen-based sedentary behavior and cognitive flexibility differs from educational sedentary behavior, providing new perspectives for fully understanding the associations between sedentary behavior and cognitive flexibility in adolescents.

## Data Availability Statement

The original contributions presented in the study are included in the article, further inquiries can be directed to the corresponding author/s.

## Ethics Statement

The studies involving human participants were reviewed and approved by the Institutional Review Board of the East China Normal University and met the guideline of the Declaration of Helsinki (No. HR047-2018). Written informed consent to participate in this study was provided by the participants’ legal guardian/next of kin.

## Author Contributions

JC and LL: conceptualization, methodology, and investigation. JC, CD, and LL: formal analysis and writing—original draft. JC and CD: writing—review and editing. All authors contributed to the article and approved the submitted version.

## Conflict of Interest

The authors declare that the research was conducted in the absence of any commercial or financial relationships that could be construed as a potential conflict of interest.

## Publisher’s Note

All claims expressed in this article are solely those of the authors and do not necessarily represent those of their affiliated organizations, or those of the publisher, the editors and the reviewers. Any product that may be evaluated in this article, or claim that may be made by its manufacturer, is not guaranteed or endorsed by the publisher.
